# Relieving distressed caregivers (ReDiCare study): study protocol of a randomized pragmatic trial

**DOI:** 10.1186/s12877-020-01941-w

**Published:** 2021-01-06

**Authors:** Klaus Pfeiffer, Christina Theurer, Gisela Büchele, Ana Babac, Helene Dick, Gabriele Wilz, the ReDiCare study group, Thomas Heidenreich, Astrid Elsbernd, Maja Reuter, Christian Ernst, Tanja Wollensak, Dietrich Rothenbacher, Maximilian Diepold, Marianna Hanke-Ebersoll, Lisa Daufratshofer, Annabella Gottswinter, A. O. K. Bavaria, Maria Gonzalez Medina, A. O. K. Baden-Wuerttemberg, Clemens Becker, Martin Hauztinger, Susanne Zank, Martina Schäufele, Frank Oswald, Sabine Jansen, Timothy Elliott

**Affiliations:** 1 Department of Clinical Gerontology and Geriatric Rehabilitation, Robert-Bosch-Hospital, Stuttgart, Germany; 2 Department of Counseling and Clinical Intervention, Friedrich Schiller University Jena, Institute of Psychology, Jena, Germany; 3 Institute of Epidemiology and Medical Biometry, Ulm University, Ulm, Germany; 4 AOK Bavaria, Munich, Germany; 5 Institute of Health Care and Public Management, University of Hohenheim, Stuttgart, Germany

**Keywords:** Caregiver, Intervention, Cognitive behavioural therapy, Problem solving, Telephone, Translation, Randomized controlled trial, Cost-effectiveness

## Abstract

**Background:**

Providing care for someone with a disease or chronic condition can have a negative psychological, physical, social, and economic impact upon informal caregivers. Despite the socio-economic relevance and more than three decades of caregiver intervention research only very few translational efforts of successful interventions are reported. Still less of these interventions have been implemented into routine services. The aim of the ReDiCare study (German acronym BerTA) is to evaluate the effectiveness of a stepped counselling approach for burdened caregivers delivered by care counsellors of two long-term care insurances and registered psychotherapists.

**Methods/ design:**

A pragmatic randomised controlled trial with 572 caregivers of older adults (≥ 60 years) receiving benefits of one of the two participating long-term care insurances. Participants are assigned (t_0_) to either the ReDiCare intervention or a control group receiving routine care and counselling. Data are collected at baseline (-t_1_), 3-month (t_1_), 9-month (t_2_) and 15-month (t_3_). The 9-month post-intervention assessment (t_2_) is the primary endpoint to evaluate the results on the primary and secondary outcomes, measured by self-reported questionnaires. Depressive symptoms measured with the CES-D are the primary outcome. The main secondary outcomes are physical complaints, utilization of psychosocial resources, caregiver self-efficacy and burden, positive aspects of caregiving and perceived care quality. A process evaluation, including audio tapes, self-report questionnaires and documentation will be conducted to examine internal and external validity of the intervention. Data on direct and indirect costs are collected for the (health) economic evaluation, using a health care perspective and a societal perspective.

**Discussion:**

While comparable previous caregiver interventions have been developed and evaluated for specific caregiver groups (e.g. dementia caregivers, stroke caregivers), the ReDiCare study will indicate whether a stepped approach will be effective also in a broader group of caregivers. The intervention is one of the very few translational studies in caregiver intervention research and will provide valuable insights into relevant factors for training, intervention protocol adherence, effectiveness, and costs for future implementation steps.

**Trial registration:**

Deutsches Register Klinischer Studien (German Clinical Trials Register), DRKS00014593 (www.drks.de, registered 14 May 2018) and International Clinical Trials Registry Platform, DRKS00014593  (https://apps.who.int/trialsearch/).

## Background

Informal caregivers, mostly family members, are an essential resource for maintaining frail elderly at home. Their typically unpaid support is a significant component of national health care systems and has gained increased research attention during the last three decades. Investigators have endeavoured to describe caregiver characteristics, their needs (e.g. for information, support, recognition) [1] and the impact of caregiving tasks on their lives. Although many caregivers report positive experiences from caregiving [2], it has been shown that providing care is commonly perceived as a chronic stressor which can cascade into mental health problems followed by possible physiological changes, impaired health habits and physical health decline [3]. Caregiver distress and burden are associated with female gender, low education, residence with the care recipient, higher number of hours spent caregiving, depression, social isolation, financial stress, and the lack of choice being a caregiver [4]. The type of disease or chronic condition of the care recipient can also impact the caregiver stress level such as dementia and cancer caregivers experience higher emotional stress than diabetes or frail elderly caregivers for example [5].

Most previous caregiver intervention research has focused on dementia caregivers. For this target group more than 200 interventions have been evaluated in randomized clinical trials during the last 35 years. Major components of these interventions have been professional support, psychoeducation, behaviour management/skill trainings, counselling/psychotherapy, self-care/relaxation training, and multicomponent interventions [6]. There is strong evidence that caregivers can benefit from cognitive behavioural therapy (CBT) and a growing trend to integrate CBT techniques into different psychoeducational programs [7]. Counselling/psychotherapy, mindfulness-based interventions, and educational programs with psychotherapeutic components appear to have the strongest effects on depressive symptoms in dementia caregivers [8]. Effective dementia caregiver interventions with small but clinically and statistically significant effects on caregiver outcomes seem to share five key characteristics [6](p. 339): active caregiver involvement in the intervention process, tailoring to specific needs identified by the caregiver, addressing different areas of need, longer-term or episodic interventions over time, and adjusting the intensity and specific focus of the intervention depending on the caregiver’s risk and need profile. Comparable general effective intervention components are also reported for interventions with other caregiver subgroups like stroke caregivers [9, 10].

Despite the social and economic relevance of caregivers for national health and long-term care systems, only very few publications on the translational process of a proven caregiver intervention have been published so far (e.g. less than 3% out of more than 200 dementia caregiver interventions previously evaluated in a RCT [11]). These few efforts have shown that complex caregiver interventions on the way into practice usually have had to be simplified to fit the delivery environment and available resources and “not the other way round” [11](S. 213). Barriers and facilitators for proven interventions on the way into practice can be due to the individuals involved (e.g. counsellors, therapists), the inner setting (e.g. compatibility of the intervention, relative priority) or the outer setting (e.g. social legislation, payment policy) [12].

In this context data on cost-effectiveness and the incremental relationship between costs and effects of different interventions are crucial for decision-makers and the implementation of new interventions into routine care (p. 11) [13]. To facilitate comparisons, health economic evaluations should be therefore based on standardized methodology [14].

In Germany, there is a growing political awareness for the nearly 2.5 million main caregivers who are the backbone of the national long-term care system [15]. Within the statutory long-term care insurance as part of the German social security system since 1995, caregiver issues have been increasingly addressed in the legislative changes of the last 10 years. Beside other beneficiaries, caregivers are entitled (with the consent of the care recipient) to information and consultation to lower their strain since 2009. In 2018 long-term care insurances spent more than 130 million Euro for care counselling (p. 49) [16] delivered by 1.332 counsellors (full-time equivalents) (p. 52). Counsellors should have a professional qualification in nursing, social work or social insurance business with an obligatory additional training (400 h) in care expertise, case management and social law according the legal requirements. Guidelines for counselling had been very general, broad, and permissive in the beginning and were somewhat specified in 2018 [17]. A recent evaluation [16] shows that a systematic training in counselling methods is lacking. Only one third of the counsellors (34%) have been specifically trained in methods to deal with problems, conflicts or challenges of caregivers and care recipients (p. 123). No data are existing on the actual use of proven counselling methods in daily routine. In a translation study based on previous research [18] we could demonstrate that using a structured method based on an initial assessment and problem-solving (PLiP intervention) can significantly reduce depressive symptoms in distressed caregivers in comparison to routine counselling [19, 20] [Pfeiffer et al. in preparation]. Despite the positive significant effect of this short intervention (one face-to-face contact and a mean of 2 follow-up contacts by phone) on caregiver depression, high depressive symptoms (CES-D ≥ 23) were still prevalent in 42% of caregivers in the intervention group after the intervention. This indicates that at least for a part of caregivers a more comprehensive and longer intervention covering more aspects of caregiving (e.g. cognitive, emotional) could have a beneficial effect.

Such more comprehensive programs for caregivers based on CBT principles and delivered by psychotherapists have been already successfully evaluated in previous efficacy [21, 22] and effectiveness studies [23–25] in Germany but have not been implemented because cost coverage by health care or long-term care insurances has not been negotiated yet.

### Objectives

In view of this background and our previous studies, the ReDiCare study combines a low-threshold short intervention (PLiP intervention) based on problem-solving with an optional telephone-based cognitive behavioural intervention in a stepped approach.

While the 3-month short intervention according to the existing care counselling scheme focusses mostly on information and everyday problem-solving, the 6-month CBT intervention addresses a broader range of cognitive and emotional consequences of caregiving. This short-term psychotherapy (12 h over 6 months) is a low-intensity manualized psychological program delivered by psychotherapists [26]. The intervention had been originally developed for dementia caregivers (Tele.TAnDem intervention) [23, 24, 27] and was extended for a broader group of caregivers regardless of the disease of the care recipient for this study (Tele.TAnDem.Plus^+^ intervention).

The main objective of this pragmatic trial is to evaluate the positive effects of this stepped intervention on depressive symptoms compared to an information-only control group receiving routine care. Main secondary caregiver outcomes are physical complaints, caregiver self-efficacy and burden, resource utilization, positive aspects of caregiving and perceived quality of care. Economic data and the incremental cost-effectiveness ratio (ICER) will be analysed to compare differences in costs and intervention effects. To identify barriers and facilitate future implementation, both interventions are embedded and delivered in routine settings. This protocol was written according to the SPIRIT 2013 reporting guidelines [28].

## Methods

### Trial design

The ReDiCare study is a pragmatic randomized controlled trial conducted in cooperation with long-term care insurances and psychotherapists working in their own practice or the psychotherapeutic ambulance of the University of Jena. The active study period (intervention and evaluation) is planned from 05/2018 to 02/2022.

### Eligibility criteria

Caregivers eligible for the trial have to provide care to a person who (a) is 60 years or older, (b) is categorised into a care degree (“Pflegegrad”) from 1 to 5 indicating the degree of self-reliance restrictions according to the German Long-Term Care Act, (c) is a member of the participating two long-term care insurances. Furthermore, participating caregivers has to (d) be at least 18 years old, (e) provide care and assistance in (instrumental) activities of daily living or supervision for ≥1.5 h per day on average or ≥ 10.5 h per week in total (including travel time), (f) have telephone access, (g) be able to communicate on the phone, and (h) report distress associated with caregiving (if two out of the following three criteria of a short screening that has been used and proven in previous studies [18, 19] are fulfilled: (1) care-related physical or mental health problems, (2) loneliness, (3) burden of care).

Caregivers are excluded when the care-recipient (a) does live in a nursing home, (b) will probably move into a nursing home within the next 3 months, (c) does receive palliative care or (d) has a terminal illness or end-stage disease with life expectancy of less than 6 months. Further exclusion criteria referring to caregivers are: (e) previous care counselling (incl. individual care plan) within the last 6 months, (f) a severe unstable or progressive disease, (g) insufficient German language skills, (h) apparent cognitive impairment, (i) a severe psychiatric diagnosis, (j) being in psychotherapy or (k) enrolled in another clinical trial for caregivers or with a psychosocial intervention.

### Intervention

Caregivers in the intervention group receive in a stepped approach a low-threshold care counselling first (intervention 1). Intervention 2 is offered depending on the counsellor’s evaluation (caregiver coping with the caregiving situation) and recommendation or, alternatively, a positive caregiver self-assessment (depressive symptoms, difficulties in coping with the caregiving situation) after t_1_. 

**Fig. 1:**
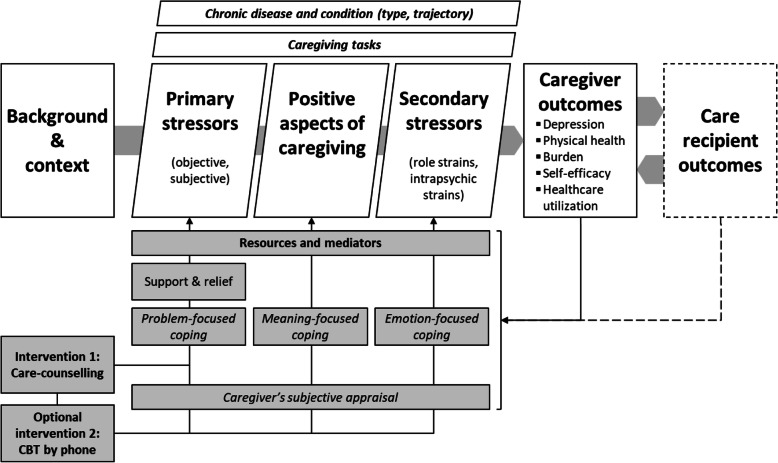
Modified model based on previous stress models [29, 32]

From the perspective of a prevalent caregiver stress-process model [29] we want to support caregiver resources and coping strategies with our intervention. Previous research could show that positive gains can coexist with stressful occurrences [30] and that benefit-finding through cognitive reappraisal can reduce depressive symptoms [31]. Therefore, we expanded Pearlin’s stress model [29] by positive aspects of caregiving (Fig. 1). We assume that increased positive emotions as outcome will in turn help to restore resources and sustain different forms of coping [32]. In line with the national care counselling guidelines [17] we address in intervention 1 mainly everyday problems and unmet practical needs of caregivers that are causing distress. The optional and more intense intervention 2 expands the focus on psychological aspects like the appraisal of the caregiving situation, emotional distress regulation (emotion-focused coping) and positive gains despite hardship (meaning-focused coping).

#### Intervention 1 (care counselling)

This short intervention is based on the problem-solving model developed by D’Zurilla and colleagues [33] and includes the following six problem-solving steps: (1) problem definition and facts, (2) optimism and orientation, (3) goal setting, (4) generation of alternatives, (5) decision making, and (6) solution implementation and verification. The six components are viewed as a continuous and interlinking process rather than stages to be followed in serial order. A caregiver burden assessment is used during the initial contact and covers 20 aspects of caregiving out of four dimensions (areas of responsibility, challenging aspects of care, subjective burden, and interaction with others). In addition, further aspects of caregiving or caregiver’s life not covered by the assessment but relevant in the current situation are asked. For each aspect, the degree of burden is rated by the caregiver on a 5-point scale ranging from "not at all burdensome" to "very burdensome". This assessment helps the caregiver to reflect the current caregiving and living situation. Possible associations of different stressful aspects are discussed and advice topics for the consultation selected.

The intervention is delivered by the two participating long-term care insurances in two different settings: (a) with an initial home visit plus telephone contacts, (b) telephone-based only. These two modalities cover both centralized and decentralized structures of the different German long-term care insurances. In the face-to-face initial contact (*setting a*) a card-sorting task covering the mentioned 20 aspects is used for the assessment. This method has been developed over years in different previous studies [18, 19, 34] and refined with counsellors from insurances for this study. Intervention materials (card assessment, three working sheets) of intervention 1 have been published by the National Association of Statutory Health Insurance Funds (GKV-Spitzenverband) in 2019 [35]. Participants of the telephone-only intervention group (*setting b*) receive a self-assessment based on the content of the cards and the same working sheets per post. In line with routine counselling data from a previous translational study [19], we defined only a minimum intensity for the intervention in both settings [20]: 1 mandatory home visit (*setting a*) or initial telephone call (*setting b*) with assessment plus at least two follow-up calls during the following 3 months (t_0_ to t_1_) and at least one more follow-up call during the next 6 months (t_1_ to t_2_) for those caregivers who do not receive the CBT intervention (intervention 2).

#### Intervention 2 (cognitive behavioural therapy)

This optional additional intervention consists of 12 (50-min) therapeutic sessions of individual CBT provided by trained psychotherapists within 6 months (t_1_ to t_2_). The first four sessions take place at weekly intervals, six further sessions will follow at fortnightly intervals and the two last sessions at monthly intervals. The original Tele.TAnDem manual was developed for dementia caregivers [26] and extended for caregivers of old and very old people with other diseases (e.g. stroke, cancer, Parkinson’s disease, incontinence). Tele.TAnDem incorporates cognitive [36], behavioral [37], problem solving [38], and emotion regulation principles for managing loss and change [39, 40] as well as resource activation [41–43].

Depending on the disease and caregiver problems, appropriate CBT strategies are described and can be selected from the comprehensive Tele.TAnDem.Plus^+^ manual. The manual consists of 10 different therapy modules which can be used and combined by the therapist in a flexible way according to the individual needs of each participant. Before starting intervention 2, the psychotherapist receive first information about the caregiver and caregiving situation from the care counsellor in a phone call. Additional phone calls between the two interventionists during intervention 2 are allowed if needed.

For the development of the intervention and study protocol care counsellors, caregivers as well as experts from the independent data monitoring and safety committee were included in different focus groups and group meetings. The major components of both interventions are summarized in Table 1, the delivery of the interventions in Table 2.

**Table 1:** The components of the stepped intervention

**Intervention 1: Care counselling** (delivered in 1 home visit/initial telephone contact and ≥ 2 follow-up phone calls by licenced care counsellors) 1. ***Caregiver assessment:*** reflecting the actual caregiving situation with burdensome aspects as well as areas of caregiving mastery, priority setting for the counselling. 2. ***Problem-solving:*** facilitating a positive problem-orientation, guided problem-solving, solution implementation, evaluation, and adaption if necessary (e.g. goal, solution, implementation plan). 3. ***Information:*** according to the national care counselling guidelines (e.g. advise on services and long-term care insurance benefits, care planning, caregiver trainings, compatibility of caregiving and work).	
**Intervention 2: Cognitive behavioural therapy** (delivered in 12 telephone contacts by psychotherapists) 1. ***Basic elements:*** creating therapeutic alliance, individual goal setting, problem analyses, resource activation, handling conflicts and crises. 2. ***Psychoeducation:*** information provision on disease related aspects (e.g. dementia) and caregiving to improve coping with caregiving (e.g. better acceptance of care recipient’s chronic condition). 3. ***Changing dysfunctional cognitions:*** developing alternative and helpful cognitions (e.g. through Socratic dialogue, guided discovery) and their implementation in daily life. 4. ***Dealing with challenging behaviour of the care recipient:*** strengthening problem-solving abilities through problem and behaviour analysis and problem-solving training. 5. ***Stress-management and emotion regulation:*** facilitating acceptance of negative emotions (e.g. anger) as normal and adaptive self-reflection from a self-distanced perspective, developing emotion-regulation strategies, working on general levels of tension and stress. 6. ***Self-care and value-based activities:*** understanding the importance of positive activities in managing mood (e.g. with a weekly diary), developing, planning, and implementing health-promoting activities in everyday life, coping with feelings like guilt as barriers for self-care (e.g. through acceptance). 7. ***Coping with change, grief, and loss:*** discussing experiences of loss and facilitating emotion-based strategies and acceptance to cope with changes caused by the care recipient’s chronic condition (e.g. changes in the relationship with the care recipient, social isolation because friends have pulled back or lack of time to spend with relatives or friends) 8. ***Informal and professional support:*** discussing possibilities of assistance and help by friends, relatives, and volunteers as well as professional services, overcoming possible barriers for accepting help (e.g. feelings of guilt) with CBT strategies like cognitive restructuring. 9. ***Limits of caregiving:*** identifying boundaries and personal limits to what can be provided at home (e.g. when caregiver stress leads to abusive behaviour, violence by the care recipient), preparing the transition from home care to formal care (e.g. nursing home). 10. ***Evaluation:*** summarizing achieved changes and goals, discussing plans and possible next steps.

**Table 2:** Delivery of the interventions (based on the TiDieR checklist [44])

	Intervention 1 (setting a, b)	Intervention 2 (optional)
WHY: rational, theory	Problem-solving	CBT, problem-solving, emotion regulation principles for managing loss and change, resource activation
WHAT: materials	a) Assessment with card sort, 3 different worksheets b) Paper-based assessment (analogue to the cards), 3 different worksheets sent by post	Assessment with Goal Attainment Scaling, different worksheets of the intervention manual sent by post.
WHO: provider	Care counsellors from long-term care insurances with different professions (social workers, nurses, social insurance employees) and an additional qualification for care counselling according to the national legal requirements. The interventions were delivered within their usual working hours. All counsellors are initially trained in a 2-day workshop by KP and supervised by psychotherapists (no members of the study team) during the study (up to one telephone contact per month). Supervisors are trained in a 1-day workshop and supervised by KP during the study when needed.	Clinical therapists with a master degree in psychology and an additional qualification in cognitive behavioural therapy. Therapists are working in different settings (psychotherapeutic outpatient clinic of the University of Jena, own practice) and are no members of the study team. They are reimbursed for all contacts provided within the study according to hourly rates for psychotherapy. All therapists are initially trained in a 1-day workshop and regularly supervised by GW during the study (4 to 6 times per year and on demand).
HOW: delivery	Individual, manual-based (short manual and materials are available online from the National Association of Statutory Health Insurance Funds) [35] (Table 1)	Individual, manual-based [26] (Table 1)
	Possible complications, ethical or legal issues arising during the study are documented and discussed with members of the advisory board (jurist, gerontopsychologist, nurse, representative of the national Alzheimer Society).	
WHERE: location	a) Initial contact in care-recipients home if possible, follow-up contacts by phone b) All contacts by phone	All contacts by phone
WHEN, HOW MUCH: dosage	a) One initial home visit, ≥ 2 contacts by phone over 3 months b) ≥ 3 contacts by phone over 3 months a+b) caregivers without Intervention 2 receive at least an additional follow-up contact during the following 6 months	12 contacts by phone over 6 months
TAILORING: what, how	Yes (assessment to identify burdensome aspects of caregiving and individual goals)	Yes (Goal Attainment Scaling to assess participants’ own, most personally relevant problems and own personal goals)
HOW WELL: delivered as planned	Evaluation based on copies of the assessment, working sheets (with goals, alternative solutions, implementation plan) and records of number and duration of contacts.	Evaluation based on therapists’ protocols from each session, GAS, records of the number and duration of contacts, protocols from supervision, audio tapes of all therapy sessions and on The Scale for the Multiperspective Assessment of General Change Mechanisms in Psychotherapy (SACiP) [45]

### Interventionists

#### Intervention 1

Interventionists are care counsellors of the two participating LTC insurances. According to the training manual, the care counsellors are trained in a two-day workshop (e.g. short presentations, role-plays, group work) with additional telephone-based training and supervision by the study team during a training period of 2 to 3 months before delivering intervention 1 in the study. After the initial training period the care counsellors are supervised by registered psychotherapists (no members of the study team) in monthly telephone contacts during their active participation in the study. Major components of these telephone calls are the delivery of the problem-solving intervention, coping with difficult interactions during counselling sessions, and mental hygiene. The last two optional aspects are included as a form of reward for participating and investing hours of work in the study. The manual for supervisors has been developed together with experts in workplace health promotion for caring professions. Supervisors of the care counsellors have received a one-day on-site training (TH from the ReDiCare study group, KP) and optional additional phone calls with the principal investigator (KP) to discuss open questions. For the intervention, 14 counsellors (home visit plus telephone / AOK Bavaria: 10 counsellors; telephone based only / AOK Baden-Württemberg: 4 counsellors) and 3 supervisors are trained. Care counsellors do not receive any extra money for participating in the study. Supervision for care counsellors is provided on a per hour fee basis for psychotherapists.

#### Intervention 2

Interventionists are CBT therapists. Based on the Tele.TAnDem.Plus^+^ manual, the CBT therapists are trained in a 1 day workshop. One part of the training focuses on the original Tele.TAnDem CBT intervention strategies [23, 26]. The other part of the training focuses on specific demands of various diseases in old age (e.g. stroke, cancer, Parkinson’s disease, and incontinence) as well as typical therapeutic problems and appropriate therapeutic strategies for these diseases and typical caregiver demands and problems. After the initial training, CBT therapists are supervised by GW in regularly group or telephone contacts (4 to 6 times per year and on demand) during the intervention period to ensure treatment integrity. Telephone therapy sessions in intervention 2 are paid on a per hour fee basis for psychotherapists. All therapy sessions are audiotaped to analyse treatment integrity.

### Control group

Participants allocated to the control group are informed about the regular care counselling offered by the insurance of the care recipient after randomisation. Each caregiver of the control group receives an allowance of 50 € for participation in the study.

### Information provision and routine care in all study groups

If a care recipient receives home care solely from informal caregivers, the federal law states that caregivers have to be visited (depending on the care level) two to four times a year by professional caregivers for information and training to ensure a sufficient quality of care. Caregivers in both conditions of this study receive this form of consultation as well as all other benefits of their health and long-term insurance. To increase the likelihood that participants of the control group remain in the study and to provide comparable general information on caregiver topics across the two states and participating LTC insurances, all participants receive nine printed information letters on caregiving (e.g. benefits of the long-term care insurance, relaxation, depression) by the study team.

### Measures

Table 3 provides an overview of the primary and secondary outcomes

**Table 3:**
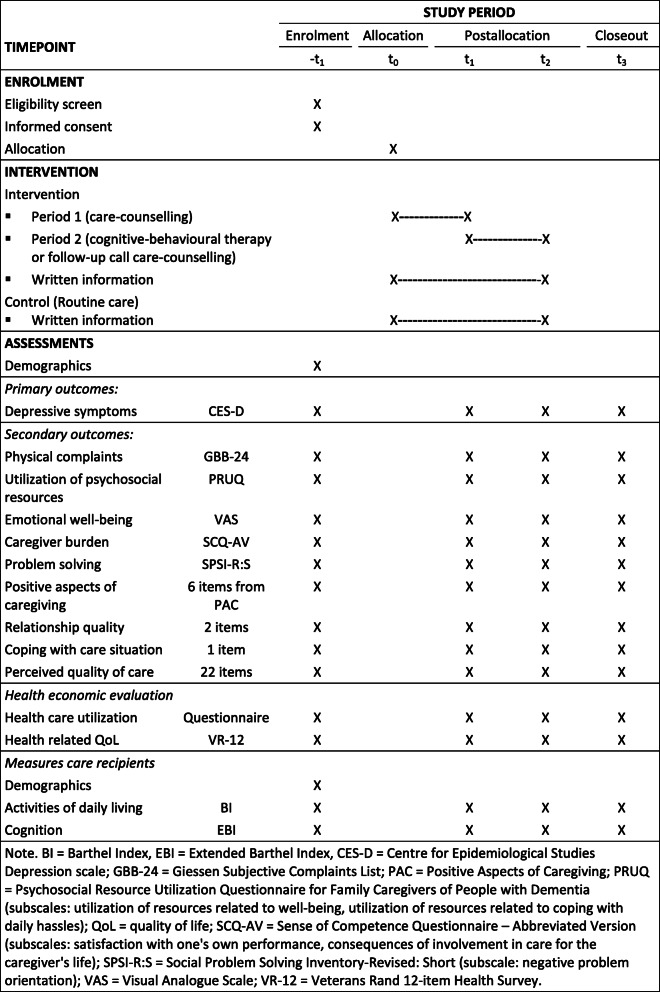
Assessments

### Primary outcome measure

#### Depressiveness

Depressive symptoms of informal caregivers are assessed by the 20-item Centre for Epidemiological Studies Depression scale (CES-D) [46, 47]. Total scores range from 0 to 60 with a score of ≥16 as an indicator of caregivers at risk for clinical depression.

### Secondary outcome measure

#### Physical complaints

Subjective physical complaints are measured by the Giessen Subjective Complaints List (GBB-24) [48]. The four domains of complaints (fatigue, stomach problems, heart problems and joint pain) are rated on a five-point scale, ranging from 0 (“not existing”) to 4 (“strong”).

#### Utilization of psychosocial resources

Two adapted subscales of the Psychosocial Resource Utilization Questionnaire for Family Caregivers of People with Dementia (PRUQ) [42] are used to measure the utilization of resources of caregivers [additional file 1]. The adapted subscales “Utilization of resources related to well-being” and “Utilization of resources related to coping with daily hassles” consist of 9 and 15 items, respectively. Participants use a 5-point scale (1 = “never”, 5 = “very often”) to indicate the frequency with which they had utilized each resource (e.g. to do something for one’s own health and physical fitness) in the last 4 weeks for achieving a motivational goal (e.g. to feel good).

#### Caregiver self-efficacy and burden

Two subscales of the abbreviated German version of the Sense of Competence Questionnaire (SCQ-AV; subscales: “Satisfaction with one’s own performance as a caregiver” and “Consequences of involvement in care for the caregiver’s life”) [49] are used in this study. Items are rated on a 5-point scale (from “agree” to “disagree”).

#### Emotional well-being

A self-developed visual analogue scale (0 to 100, vertical) is used to assess emotional well-being [24, 27].

#### Coping with problems

The subscale “Negative Problem Orientation” of the Social Problem Solving Inventory – Revised Quik Score form (SPSI-R:S) [50] [51] is used to assess negative problem orientation. This subscale contains five items that are rated on a 5-point scale ranging from 0 (“not very true of me”) to 4 (“extremely true of me”). The total score ranges from 0 to 20.

#### Coping with burden of care

Based on the experience of our previous studies a single item (“How well are you able to cope with the care situation?”; from 0 (“very badly”) to 4 (“very well”) is used to measure coping with burden of care [24].

#### Positive aspects of caregiving

Psychosocial benefits of caregiving among family caregivers are assessed with six Items (Items 2, 3, 4, 7, 8, 9) of the Positive Aspects of Caregiving (PAC) scale [52]. Items are scored on a 5-point scale from 1 (“disagree a lot”) to 5 (“agree a lot”).

#### Perceived quality of care and abusive behaviour

A self-developed questionnaire with 22 items measures different indicators of quality of care (e.g. health care, nursing, environment, decubitus, forms of harmful behaviour and abuse by the caregiver). Items are rated on a 5 point-scale from 0 to 4 with different categories (e.g. “never” to very often / 3–7 times per week) (additional file 2).

#### Quality of relationship

The current and past quality of relationship is measured with two questions (“What is the quality of your current relationship with your relative?” and “What was the quality of your relationship with your relative before he/she needed care?”). Both questions are rated on a 5-point scale from 0 (“very bad”) to 4 (“very good”).

### Other measures

#### Caregiver characteristics

For caregivers, detailed conditions of the care situation, diseases, sleep quality, and health service use are assessed [23, 24].

#### Activities of daily living and cognitive functioning

For the care recipient characteristics, type of diseases, functional disability, health and care service use are assessed. Activities of daily living (ADL) are measured with the Barthel Index (BI) [53], higher cognitive functioning (comprehension, verbal expression, social interaction, problem solving, memory/learning/orientation, and vision/neglect) with the Extended Barthel Index (EBI) [54].

#### Cost effectiveness

The cost-effectiveness analysis is conducted from a health care perspective and a societal perspective with a time horizon of 3 to 12 months. The costs are recorded on the basis of statutory health insurance data and data provided by the caregivers. Additional information on non-reimbursed caregiver time and out-of-pocket expenditures are collected (e.g. types of daily assistance). Collection of cost data will follow established international standards using the costing methodology suggested by Drummond [13]. If participants agree, available data on medical resource utilization from the two cooperating statutory health insurances are included in the analyses. As a measure of effectiveness, subjective health-related quality of life is measured with the Veterans Rand 12-item Health Survey (VR-12) [55, 56]. Items of the VR-12 are summarized in two scores, a physical and mental component score. To perform the cost-effectiveness analysis, the incremental cost-effectiveness ratio (ICER) will be calculated, comparing the costs and outcomes of different modalities (interventions, routine care).

#### Satisfaction of patients and interventionists with the intervention

A set of questions is used to assess the satisfaction of caregivers with intervention 1 at t_1_, and with intervention 2 at t_2_. Six questions cover the general setup of the intervention (setting, number of sessions, and duration of a single session) and perceptions of helpful and unhelpful aspects of the intervention, further 24 items the competence and experience with the counsellor and therapist. In addition, three items of the Client Satisfaction Questionnaire (CSQ-8) [57] focus on the quality of service, and general satisfaction of the participant. After the follow-up (t_3_) the participants are asked to comment on the intervention on ten different aspects (ratings and open questions) from a retrospective perspective. In addition to the caregivers, all care counsellors in intervention 1 are asked for feedback (ratings and open questions) on their experience with the intervention approach (materials, feasibility, etc.), the training, supervision and supervisors after the initial training, the middle and end of the intervention period.

In intervention 2 Goal Attainment Scaling (GAS) [58] is used to evaluate participants’ personal goal attainment. During the first and/or second telephone sessions, the caregiver and the therapist identify the caregiver’s personal goals for the intervention 2. The steps of GAS are formulating goals, describing goals in observable terms, defining a hierarchy of goals and potential outcomes. In the last therapy session, therapists and caregivers rate the goal attainment.

### Process evaluation

Major goals of the process evaluation are to document how well the intervention was implemented as designed (domain 1 “Implementation”), to describe how caregivers participated in and responded to the intervention, including any variations across the two settings in intervention 1 (domain 2 “Participation and response”), and to describe and explore contextual factors that may influence the delivery or impact of the intervention (domain 3 “Context”) [59].

#### Intervention 1

Documentation of all contacts with caregivers by standardized documentation sheets (date, session duration, mode of contact, distance and travel time for home visits). To assess treatment fidelity, worksheets with information on key components of intervention 1 (selected goal(s), alternative solutions, the solution plan and goal attainment) are rated by independent raters. To control for effects related to counsellor characteristics, further counsellor data like the professional background (e.g. previous professional trainings, professional experience), motivation to participate in the study, the appraisal of the intervention, number of consultations within the study, counsellor self-efficacy, work-related sense of coherence, chronic stress, and compassion are evaluated.

#### Intervention 2

Implementation is assessed by standardized documentation sheets. Therapists record session date, session duration, if and why an appointment was changed, if and why a session was interrupted, and intervention and/or study drop-out. To assess treatment compliance, therapists use standardized documentation sheets to note session content, to rate main themes and intervention strategies of the session.

The Scale for the Multiperspective Assessment of General Change Mechanisms in Psychotherapy (SACiP) [45] is used to assess the mechanism of change from both therapists’ and patients’ perspectives. Therapists and caregivers rate the six scales (agreement on collaboration, resource activation, problem actuation, clarification of meaning, emotional bond and mastery) after each telephone session.

Patients’ expectations regarding therapy and outcome are measured by the “Patient Questionnaire on Therapy Expectation and Evaluation” (PATHEV) [60] before session 1 and after session 10. This questionnaire consists of 11 items and three subscales: hope of improvement, fear of change, and suitability. Treatment integrity, such as therapists’ adherence and competence, will also be judged by independent raters, who will be qualified to rate treatment adherence and competence by completing an intensive training, during which the therapy manual and the application of the rating scales will be explained. Audiotapes from the intervention 2 will be randomly selected for treatment integrity rating. The items developed for the adherence scale are based on the intervention modules described in the manual. Conceptualization of the competence scale is based on our previous studies [23, 61, 62].

### Participant timeline

**Fig. 2:**
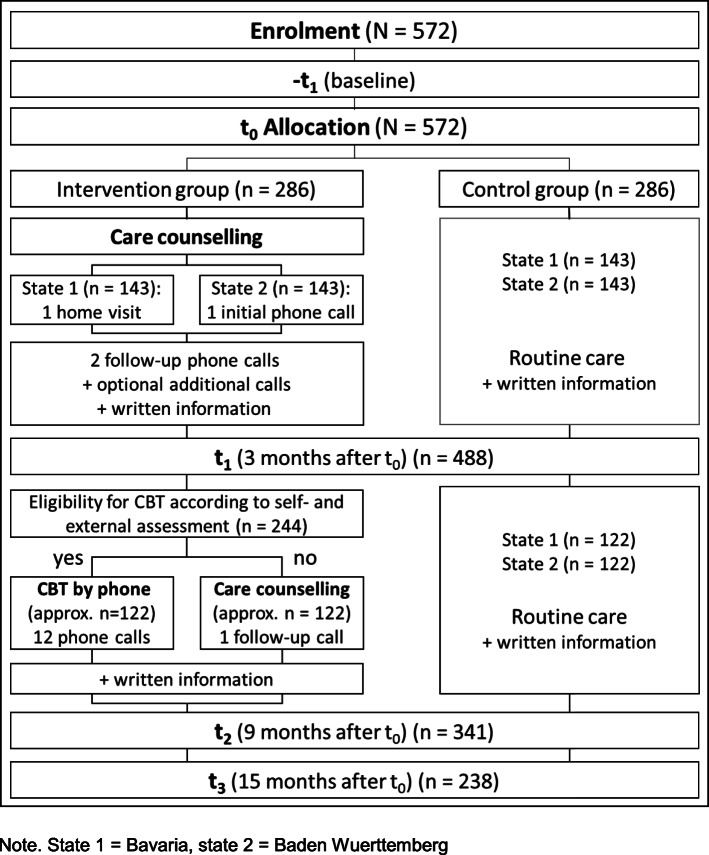
Study design and planned number of participants at each stage. Note. State 1 = Bavaria, state 2 = Baden- Württemberg

After enrolment, all participants are assessed (-t_1_) and assigned (t_0_) to either the intervention or control arm of the study. During the first 3 months participants of the intervention group receive intervention 1 (care counselling). During the next 6 months caregivers in the intervention group receive CBT (intervention 2) over 6 months or, alternatively, a follow-up call delivered by the counsellor of intervention 1. All outcomes are assessed again after 3 months (t_1_), 9 months (t_2_), and 15 months (t_3_) (Fig. 2).

If caregiving ends during the intervention period (e.g. because of death of care recipient, transition to nursing home) we offer a (continuation) of the CBT intervention based on a case-by-case decision. All participants who end caregiving before t_3_ are followed up with a modified questionnaire. These post-caregiving data will be not included in the main outcome analysis.

### Sample size

Sample size calculation is based on analyses of covariance (ANCOVA) to compare post-interventions scores of both groups at t_2_ using -t_1_ scores as independent variables. The sample size for the ANCOVA was calculated in a two-step method as proposed by Borm et al. [63]: (1) Calculation of the sample size n as if a t-test on the follow-up scores was carried out, (2) the number of subjects was then multiplied by a “design factor” [(1 – ρ^2^)*n; ρ = correlation between baseline and follow-up scores] to produce the number of subjects required for the ANCOVA.

The calculation is based on data of previous studies [18, 19, 24] with informal caregivers and the following assumptions: (a) An aimed relevant improvement of the CES-D scores of 3 points compared to the control condition (corresponding effect size Cohen’s d of 0.34); (b) depressive symptoms at t_2_ (control: M = 21.0, SD = 10.1; intervention: M = 18.0, SD = 7.6); (c) a correlation ρ = 0.49 between baseline and follow-up measures (the lowest ρ was used); (d) 1- β = .80 (power); and (e) α = .05 for a two-sided t test, (f) a ratio of n (control) / n (intervention) = 1.00. (1) Using a formula for a two-sample t-test (G*Power Vs. 3.1.9.2) [64] the required total sample size is 282. (2) The required adjusted sample size for ANCOVA with the same power as the t-test is 216 [design factor: (1- 0.49^2^)*n (total)].

Possible different strategies to promote study participation of the two participating insurances are an element of uncertainty in the sample size calculation. Therefore, we added an additional 10% to the calculated 216 participants to compensate a possible shift in variance. Taking this into account, the aimed sample size is 238.

Based on the same assumptions (two-sided t-test, α = .05, 1- β = .80, d = .34) and data of our previous work, the correlations between baseline and follow-up measures of secondary outcomes are expected to be ρ = .40 (VAS Mood), ρ = 0.72 (GBB-24), 0.59 (SPSI-R:S / Subscale “Negative problem orientation”), and ρ = 0.70 (SCQ-AB / Subscale “satisfaction with one’s own performance”). Because of these higher correlations in comparison to the CES-D we assumed that the total sample size of 216 participants will be sufficient to detect at least moderate effects in all secondary outcome measures except the ‘VAS Mood’. For this scale, the aimed sample size of 238 is still sufficient. 

Based on our previous studies we assumed dropout rates of 15% between -t_1_ and t_1_ and 20% in each of the following two 6-month intervals (t_1_ to t_2_, t_2_ to t_3_). Based on these assumptions we aim to enrol 572 caregivers. The participant flow and numbers through the study are summarized in Fig. 2.

### Recruitment

Recruitment including a short screening for caregiver burden (eligibility criteria h) is carried out by employees of two participating German long-term care insurances (AOK Baden-Württemberg, AOK Bavaria). These two insurances have together 8.4 million members with more than 350,000 of them being dependent on care. Different recruitment strategies are planned to get access to the target group [e.g., caregivers of care recipients after an increase of the care degree (§18 SGB XI), mandatory counselling (§37.3 SGB XI), contacting insurance members via phone, letters and other forms of advertising]. The planned strategies are coordinated with the administration and data protection officials of the insurances. Each of the two participating insurances is planned to recruit 50% (*N* = 286) of the aimed total number of participants. Employees of the insurances inform caregivers about the study, administer the short screening (eligibility criteria “h”) to identify distressed caregivers, and ask for the consent to forward the caregiver contact details to the study team of the University of Jena. A member of the study team informs about further details of the study (via phone and written information) to enable the caregiver to make an informed decision whether to participate in the study or not. Participants have to agree to written and audiotaped (only intervention 2) records of the sessions, trial-related audits, readout of health care cost-data if being a member of one of the two participating insurances (2 years before -t_1_ until t_3_), and data processing and analyses within the study. The signed consent form has to be sent in a prepaid reply envelope to the study team of the University of Jena before enrolment.

### Allocation

After baseline assessment (-t_1_) caregivers are randomly assigned (t_0_) to one of two groups (intervention or usual care control group). Concealed randomization sequence is performed in permuting blocks with different block sizes. The allocation ratio is 1:1. Randomization is stratified for region respective insurance (AOK Bavaria, AOK Baden-Württemberg with a 1:1 ratio) and for sex (male, female with 1:4 ratio). The computer-generated randomization lists have been generated by an independent randomization unit at the University of Ulm. After receiving a new ID number including sex and state via E-Mail from the study team of the University of Jena, the allocation is performed and mailed back by a study assistant from the University of Ulm not involved in the recruitment processes and without any participant contacts.

### Blinding

In accordance with the requirements of the data protection guidelines of the two insurances, the scientific partners and the ministries of social affairs of the two states, study participants have to be informed about the different possible study arms and interventions before enrolment. Therefore, participating caregivers are not blinded. Counsellors of the insurances and the psychotherapists delivering the interventions are informed that they provide an intervention within the ReDiCare study. Participants should complete questionnaires themselves. When the blinded assessors are trying to clarify open questions and checking the completeness of the questionnaires in a phone call, participants can reveal their intervention. Data analysts of the University of Ulm are blinded to the allocation status.

### Data collection methods

For assessments, the evaluation team (psychologists) send the questionnaires with individual code numbers by post to the caregivers who are asked to answer the questions. In a following phone contact the questionnaire is reviewed for completeness and unclear points are discussed. After this telephone contact, the questionnaires should be returned in a return envelope. This survey technique with an additional phone call is used because a mostly elderly study population is expected and, in particular, the questions on the used health services are often not easily answered. Furthermore, the acceptance of the survey is to be improved and the risk of missing data or dropouts to be reduced.

### Data management

Original questionnaires are scanned, classified, recognized, verified by the study team at the University of Jena and transferred with the TeleForm®-software (version 10.8, Electric Paper Informationssysteme GmbH, Lüneburg, Germany) into the data base of the data centre of the University of Ulm. All data communication processes, data storage and encoding are specified in detail in a data protection contract according to the European General Data Protection Regulation. Data preparation and statistical analyses will be performed with SPSS (IBM Corp., Armonk, NY), SAS (SAS Institute Inc., Cary, NC, USA) and MPlus (Muthén & Muthén, Los Angeles, CA) at Ulm University.

### Statistical methods

Comparability in all baseline parameters of the two randomized study groups will be described. For the intention-to-treat (ITT) analysis, missing values for withdrawn subjects or participants with missing data at t_1_ to t_3_ will be imputed by MLMI (Maximum Likelihood Multiple Imputation). For each outcome, ten data sets with imputed values (based on linear regression prediction) will be generated and the results pooled to obtain final estimates.

Inference statistic will be performed according to the variable type with χ^2^-test or Fisher exact tests for categorical variables, Mann–Whitney U tests for ordinal or not normally distributed continuous variables, and t-tests for normally distributed continuous variables. Group differences of the confirmatory endpoints will be tested with ANCOVAs (Analyses of Covariance) considering relevant baseline covariates on a two-sided significance level of 5% taking multiple testing into account. Effects of the intervention on secondary outcomes will be analysed accordingly. Additionally, random coefficient models and latent change analyses will be applied to estimate group effects in longitudinal data structures with repeated measurements. The health economic evaluation will include analyses of intervention costs, cost-effectiveness, and possible cost-benefits.

### Data monitoring

The independent data monitoring and safety committee is composed by experts in counselling and CBT interventions, gerontology, caregiver issues, elder abuse and personal rights. All members (see acknowledgement) are independent from the funder and competing interests. They will also be convened to detect any trends, such as increases in un/expected events, and take appropriate action, to seek additional advice or information from investigators where required, to evaluate the risk of the trial continuing and take appropriate action where necessary. The whole committee is planned to meet once a year. Specific experts can be contacted as needed.

### Harms

Adverse events of the intervention or assessment procedures are not expected. However, any adverse events or misconducts of interventionists are monitored throughout the trial. All data are logged and any serious adverse events are reported to the independent data monitoring and safety committee. In this case the committee will discuss any systematic adverse effects, suggest modifications in the protocol or the termination of the trial.

### Auditing

An independent data manager of the Centre for Clinical Studies of the Jena University Hospital will monitor the current study according the Quality Management and Monitoring Plan (e.g. checking completeness and correctness of study documents, study data, and data input) every 4–5 months.

### Protocol amendments

Because dropout rates have been lower than expected in this ongoing study, the aimed recruitment number has been reduced from 572 to 504 to achieve the planned study completer sample of 238 caregivers. In line with the changing actual national and local COVID-19 restrictions and official instructions of the participating long-term care insurances (restricted home visits), the following alternative settings for the initial home visit in *setting a* of intervention 1 are allowed: face-to-face contact in the service centre of the long-term care insurance or telephone-based initial contact. These possible setting variations because of actual restrictions will be considered in the data analyses.

## Discussion

This study aims to evaluate the effectiveness of a stepped counselling approach for burdened caregivers delivered by care counsellors of two long-term care insurances and registered psychotherapists. This stepped intervention approach can be tailored to specific caregiver’s risk and need profile (e.g. practical needs, emotions) in a resource efficient way. In contrast to most previous interventions that have been evaluated for specific caregiver subgroups (e.g. dementia-caregivers, stroke-caregivers, etc.) we include distressed caregivers of older adults with any chronic condition. With this broader target group, we assume to facilitate the uptake of the intervention in settings providing counselling services for all types of caregivers. The possibility to deliver the whole intervention by phone expands the access for caregivers in rural areas and facilitates future services in minority languages.

Furthermore, the ReDiCare study is one of the very few translational studies in caregiver intervention research and allows to evaluate the effectiveness of the intervention in real-world practice settings. With the randomized controlled design and the 6-month follow-up we also address methodological limitations of most previous translational studies in this field [11].

There are also limitations in this study. Because of the required informing of the participants in this pragmatic trial the participants are aware in which group they are allocated. Improvements of the intervention group can be biased from factors like motivation and expectations regarding the intervention (placebo), social contact with the interventionist, etc. Interventionists are also be aware that their interventions are evaluated and compared with other conditions. As in other psychosocial trials, the blinding of assessors is also questionable. When checking the caregiver assessments for unclear points and missing responses, participants can reveal information on the interventions that will allow the assessor to identify the treatment condition. While we include more types of caregivers than most other caregiver intervention studies, caregivers of adults aged less than 60 years are excluded due to the call for this proposal.

Despite these limitations, delivering the intervention within routine care settings will provide valuable insights not only on effectiveness but also on costs and crucial factors for training, maintaining, and implementing the two interventions. Therefore, we hope that the results of the ReDiCare trial will contribute to stimulate further implementation efforts in this field. The findings of the current study will be published in peer-reviewed journals and presented at scientific conferences. Besides the scientific dissemination, we want to present and discuss the results with decision makers, insurances, NGOs and caregivers’ representatives in a public final meeting and other settings.

## Data Availability

Materials and major components of the two interventions are published in German [26, 35]. Further information is available from KP and GW on reasonable request. This study protocol does not contain any data or results of the evaluation. Scientific trial members of the University of Jena, the Robert-Bosch-Hospital, the University of Hohenheim (health economic data), and the study statistician from the University of Ulm will have access to the final data set. To ensure confidentiality, data distributed to members of the study team will be blinded of any identifying participant information.

## Abbreviations

ADL: Activities of daily living
ANCOVA: Analysis of covariance
AOK: Local Health Insurance Fund
BerTA: Beratung und telefonische Therapie für pflegende Angehörige [counselling and telephone-based therapy for caregivers]
BI: Barthel Index
CBT: Cognitive behavioural therapy
CES-D: Center for Epidemiologic Studies Depression Scale
CSQ-8: Client Satisfaction Questionnaire
EBI: Extended Barthel Index
GAS: Goal Attainment Scaling
GBB-24: Giessen Subjective Complaints List
ICER: Incremental cost-effectiveness ratio
ITT analysis: Intention-to-treat analysis
LTC insurances: Long-term care insurances
MLMI: Maximum Likelihood Multiple Imputation
PAC: Positive Aspects of Caregiving
PATHEV: Patient Questionnaire on Therapy Expectation and Evaluation
PLiP: ProblemLösen in der Pflegeberatung [problem-solving in care counselling]
PRUQ: Psychosocial Resource Utilization Questionnaire for Family Caregivers of People with Dementia
RCT: Randomized controlled trial
ReDiCare: Relieving Distressed Caregivers
SACiP: Scale for the Multiperspective Assessment of General Change Mechanisms in Psychotherapy
SCQ-AV: Sense of Competence Questionnaire – Abbreviated Version
SGB: Social act
SPSI-R:S: Social Problem Solving Inventory – Revised: Short
State 1: Bavaria
State 2: Baden-Württemberg
Tele.TAnDem: Cognitive-behavioural therapy telephone-based intervention for family caregivers of people with dementia
TIDieR: Template for Intervention Description and Replication Checklist and Guide
-t_1_: Baseline assessment
t_0_: Allocation after -t_1_
t_1_: Assessment 3 months after t_0_
t_2_: Assessment 6 months after t_1_ (post-intervention)
t_3_: Assessment 6 months after t_2_ (follow-up)
VAS: Visual Analogue Scale
VR-12: Veterans Rand 12-item Health Survey

## References

[1] Plöthner M, Schmidt K, de Jong L, Zeidler J, Damm K (2019). Needs and preferences of informal caregivers regarding outpatient care for the elderly: a systematic literature review. BMC Geriatr.

[2] Roth DL, Fredman L, Haley WE (2015). Informal caregiving and its impact on health: a reappraisal from population-based studies. The Gerontologist.

[3] Schulz R, Sherwood PR (2008). Physical and Mental Health Effects of Family Caregiving: AJN. Am J Nurs.

[4] Adelman RD, Tmanova LL, Delgado D, Dion S, Lachs MS (2014). Caregiver burden: a clinical review. JAMA..

[5] Kim Y, Schulz R (2008). Family caregivers’ strains: comparative analysis of Cancer caregiving with dementia, diabetes, and frail elderly caregiving. J Aging Health.

[6] Gitlin L, Hodgson N, Gaugler JE, Kane RL (2015). Caregivers as therapeutic agents in dementia care: the context of caregiving and the evidence base for interventions. Family caregiving in the new Normal.

[7] Cheng S-T, Au A, Losada A, Thompson LW, Gallagher-Thompson D (2019). Psychological interventions for dementia caregivers: what we have achieved, What We Have Learned. Curr Psychiatry Rep.

[8] Cheng S-T, Li K-K, Losada A, Zhang F, Au A, Thompson LW (2020). The effectiveness of nonpharmacological interventions for informal dementia caregivers: an updated systematic review and meta-analysis. Psychol Aging.

[9] Bakas T, Clark PC, Kelly-Hayes M, King RB, Lutz BJ, Miller EL (2014). Evidence for stroke family caregiver and dyad interventions: a statement for healthcare professionals from the American Heart Association and American Stroke Association. Stroke J Cereb Circ.

[10] Bakas T, McCarthy M, Miller ET. Update on the state of the evidence for stroke family caregiver and dyad interventions. Stroke. 2017;48. 10.1161/STROKEAHA.117.016052. 10.1161/STROKEAHA.117.016052 PMC5404967 28351961

[11] Gitlin LN, Marx K, Stanley IH, Hodgson N (2015). Translating evidence-based dementia caregiving interventions into practice: state-of-the-science and next steps. The Gerontologist.

[12] Damschroder LJ (2020). Clarity out of chaos: use of theory in implementation research. Psychiatry Res.

[13] Drummond MF, Sculpher MJ, Claxton K, Stoddart GL, Torrance GW (2015). Methods for the economic evaluation of health care programmes.

[14] Bongiovanni-Delarozière I, Le Goff-Pronost M (2017). Economic evaluation methods applied to telemedicine: from a literature review to a standardized framework. Eur Res Telemed Rech Eur En Télémédecine.

[15] Wetzstein M, Rommel A, Lange C. Informal caregivers - Germany’s largest nursing service. Robert Koch-Institut. 2016. 10.25646/3065.

[16] Wolf JK, Pflug C, Relleck J, Rieckhoff S, Dehl T, Nolting H-D. Evaluation der Pflegeberatung und Pflegeberatungsstrukturen gemäß § 7a Absatz 9 SGB XI. [Evaluation of care counselling and care counselling structures according §7a section 9 social care act XI]. Abschlussbericht [Final report]. Berlin: IGES Institut; 2020. www.gkv-spitzenverband.de/media/dokumente/pflegeversicherung/beratung_und_betreuung/pflegeberatung/20200331_IGES_Evaluation_Pflegeberatung_Abschlussbericht.pdf. Accessed 1 Sep 2020.

[17] GKV-Spitzenverband, editor. Richtlinien des GKV-pitzenverbandes zur einheitlichen Durchführung der Pflegeberatung nach § 7a SGB XI vom 7. Mai 2018 (Pflegeberatungs-Richtlinien). 2018. https://www.gkv-spitzenverband.de/media/dokumente/pflegeversicherung/richtlinien__vereinbarungen__formulare/richtlinien_zur_pflegeberatung_und_pflegebeduerftigkeit/180531_Pflegeberatungs-Richtlinien_7a_SGB_XI.pdf. Accessed 1 Sep 2020.

[18] Pfeiffer K, Beische D, Hautzinger M, Berry JW, Wengert J, Hoffrichter R (2014). Telephone-based problem-solving intervention for family caregivers of stroke survivors: a randomized controlled trial. J Consult Clin Psychol.

[19] Pfeiffer K, Hautzinger M, Patak M, Grünwald J, Becker C, Albrecht D (2017). Problem-solving in caregiver-counselling (PLiP study): study protocol of a cluster randomized pragmatic trial. BMC Geriatr.

[20] GKV-Spitzenverband, editor. ProblemLösen in der Pflegeberatung - ein Ansatz zur Stärkung der Pflegeberatung nach § 7a SGB XI. Hürth: CW Haarfeld.

[21] Wilz G, Soellner R. Evaluation of a Short-Term Telephone-Based Cognitive Behavioral Intervention for Dementia Family Caregivers. Clinical Gerontologist. 2015;39(1):25-47. 10.1080/07317115.2015.1101631.

[22] Wilz G, Meichsner F, Soellner R (2017). Are psychotherapeutic effects on family caregivers of people with dementia sustainable? Two-year long-term effects of a telephone-based cognitive behavioral intervention. Aging Ment Health.

[23] Soellner R, Reder M, Machmer A, Holle R, Wilz G. The Tele.TAnDem intervention: study protocol for a psychotherapeutic intervention for family caregivers of people with dementia. BMC Nurs. 2015;14. 10.1186/s12912-015-0059-9 PMC5395922 28428730

[24] Wilz G, Reder M, Meichsner F, Soellner R (2018). The Tele.TAnDem Intervention: Telephone-based CBT for Family Caregivers of People With Dementia. The Gerontologist.

[25] Meichsner F, Töpfer NF, Reder M, Soellner R, Wilz G (2019). Telephone-Based Cognitive Behavioral Intervention Improves Dementia Caregivers’ Quality of Life. Am J Alzheimers Dis Dementias®.

[26] Wilz G, Schinköthe D, Kalytta T (2015). Therapeutische Unterstützung für pflegende Angehörige von Menschen mit Demenz: das Tele.TAnDem-Behandlungsprogramm [Therapeutic support for dementia caregivers: the Tele.TAnDem treatment programme].

[27] Wilz G, Soellner R (2016). A randomized controlled trial of cognitive behavioral therapy (CBT) for family caregivers of people with dementia: does a short term intervention on telephone work?. Clin Gerontol.

[28] Chan A-W, Tetzlaff JM, Altman DG, Laupacis A, Gøtzsche PC, Krleža-Jerić K (2013). SPIRIT 2013 statement: defining standard protocol items for clinical trials. Ann Intern Med.

[29] Pearlin LI, Mullan JT, Semple SJ, Skaff MM (1990). Caregiving and the stress process: an overview of concepts and their measures. The Gerontologist.

[30] Cheng S-T, Mak EPM, Lau RWL, Ng NSS, Lam LCW (2016). Voices of Alzheimer caregivers on positive aspects of caregiving. The Gerontologist.

[31] Cheng S-T, Mak EPM, Fung HH, Kwok T, Lee DTF, Lam LCW (2017). Benefit-finding and effect on caregiver depression: a double-blind randomized controlled trial. J Consult Clin Psychol.

[32] Folkman S (2008). The case for positive emotions in the stress process. Anxiety Stress Coping.

[33] D’Zurilla TJ, Nezu AM, Maydeu-Olivares A, Chang E, D’Zurilla TJ, Sanna LJ (2004). Social problem solving: theory and assessment. Social problem solving: theory, research and training.

[34] Dautel A, Eckert T, Gross M, Hauer K, Schäufele M, Lacroix A (2019). Multifactorial intervention for hip and pelvic fracture patients with mild to moderate cognitive impairment: study protocol of a dual-Centre randomised controlled trial (OF-CARE). BMC Geriatr.

[35] GKV-Spitzenverband. Zusatzmaterialien zum PLiP-Kartenset [Additional materials to the PLiP card set]. Pflegeberatung nach § 7a SGB XI [Care counselling according to § 7a Social Care Act XI). https://www.gkv-spitzenverband.de/pflegeversicherung/beratung_und_betreuung/pflegeberatung/pflegeberatung.jsp. Accessed 1 Sep 2020.

[36] Beck AT, Rush AJ, Shaw BF, Emery G (1979). Cognitive therapy of depression.

[37] Lewinsohn PM, Graf M (1973). Pleasant activities and depression. J Consult Clin Psychol.

[38] D’Zurilla TJ, Goldfried MR (1971). Problem solving and behavior modification. J Abnorm Psychol.

[39] Meichsner F, Schinköthe D, Wilz G (2016). The caregiver grief scale: development, exploratory and confirmatory factor analysis, and validation. Clin Gerontol.

[40] Meichsner F, Wilz G (2018). Dementia caregivers’ coping with pre-death grief: effects of a CBT-based intervention. Aging Ment Health.

[41] Grawe K (1997). Research-informed psychotherapy. Psychother Res.

[42] Töpfer NF, Wilz G (2018). Tele.TAnDem increases the psychosocial resource utilization of dementia caregivers. GeroPsych..

[43] Töpfer NF, Wilz G. Increases in utilization of psychosocial resources mediate effects of cognitive-behavioural intervention on dementia caregivers’ quality of life. J Posit Psychol. 2020:1–11.

[44] Hoffmann TC, Glasziou PP, Boutron I, Milne R, Perera R, Moher D (2014). Better reporting of interventions: template for intervention description and replication (TIDieR) checklist and guide. BMJ.

[45] Mander JV, Wittorf A, Schlarb A, Hautzinger M, Zipfel S, Sammet I (2013). Change mechanisms in psychotherapy: multiperspective assessment and relation to outcome. Psychother Res.

[46] Radloff LS (1977). The CES-D scale: a self-report depression scale for research in the general population. Appl Psychol Meas.

[47] Hautzinger M, Bailer M, Hofmeister D, Keller F (2012). Allgemeine Depressionsskala (ADS). Psychiatr Prax.

[48] Braehler E, Scheer JW (2008). Gießener Beschwerdebogen [Giessen Subjective Complaints List] (GBB) Manual.

[49] Pendergrass A, Beische D, Becker C, Hautzinger M, Pfeiffer K (2015). An abbreviated German version of the sense of competence questionnaire among informal caregivers of relatives who had a stroke: development and validation. Eur J Ageing.

[50] D’Zurilla TJ, Nezu AM, Maydeu-Olivares A (2002). Social problem-solving inventory-revised (SPSI-R).

[51] Graf A (2003). Psychometrische Überprüfung einer deutschsprachigen Übersetzung des SPSI-R. Z Differ Diagn Psych.

[52] Tarlow BJ, Wisniewski SR, Belle SH, Rubert M, Ory MG, Gallagher-Thompson D (2004). Positive aspects of caregiving: contributions of the REACH project to the development of new measures for Alzheimer’s caregiving. Res Aging.

[53] Mahoney FJ, Barthel DW (1965). Functional evaluation: the Barthel-index. Md Med J.

[54] Prosiegel M, Böttger S, Schenk T, König N, Marolf M, Vaney C (1996). Der erweiterte Barthel-Index (EBI) - eine neue Skala zur Erfassung von Fähigkeitsstörungen bei neurologischen Patienten. [The extended Barthel-Index (EBI): a new scale to assess disability in neurological patients]. Neurol Rehabil.

[55] Kazis LE, Lee A, Spiro AI, Rogers W, Ren XS, Miller DR (2004). Measurement comparisons of the medical outcomes study and veterans SF-36 health survey. Health Care Financ Rev.

[56] Buchholz I, Kohlmann T, Buchholz M. Vergleichende Untersuchung der psychometrischen Eigenschaften des SF-36/SF-12 vs. VR-36/VR-12 [A comparison of the psychometric properties of the SF-36/SF-12 and VR-36/VR-12]. Abschlussbericht [Final report]. Verein zur Förderung der Rehabilitationsforschung in Hamburg, Mecklenburg-Vorpommern und Schleswig-Holstein e.V.; 2017. https://www.reha-vffr.de/images/vffrpdf/projekte/2017/VR-Abschlussbericht_vffr205.pdf.

[57] Attkisson CC, Zwick R (1982). The client satisfaction questionnaire. Eval Program Plann.

[58] Wilz G, Weise L, Reiter C, Reder M, Machmer A, Soellner R (2018). Intervention Helps Family Caregivers of People With Dementia Attain Own Therapy Goals. Am J Alzheimers Dis Dementias®.

[59] Haynes A, Brennan S, Carter S, O’Connor D, Schneider CH, Turner T (2014). Protocol for the process evaluation of a complex intervention designed to increase the use of research in health policy and program organisations (the SPIRIT study). Implement Sci IS.

[60] Schulte D (2005). Messung der Therapieerwartung und Therapieevaluation von Patienten (PATHEV). Z Für Klin Psychol Psychother.

[61] Schinköthe D, Wilz G (2014). The assessment of treatment integrity in a cognitive behavioral telephone intervention study with dementia caregivers. Clin Gerontol.

[62] Schinköthe D, Altmann U, Wilz G (2015). The effects of treatment adherence and treatment-specific therapeutic competencies on outcome and goal attainment in telephone-based therapy with caregivers of people with dementia. Aging Ment Health.

[63] Borm GF, Fransen J, Lemmens WA (2007). A simple sample size formula for analysis of covariance in randomized clinical trials. J ClinEpidemiol.

[64] Faul F, Erdfelder E, Lang A-G, Buchner A (2007). G*power 3: a flexible statistical power analysis program for the social, behavioral, and biomedical sciences. Behav Res Methods.

